# Src-dependent phosphorylation at Y406 on the thyroid hormone receptor β confers the tumor suppressor activity

**DOI:** 10.18632/oncotarget.2487

**Published:** 2014-09-16

**Authors:** Jeong Won Park, Li Zhao, Paul Webb, Sheue-yann Cheng

**Affiliations:** ^1^ Laboratory of Molecular Biology, Center for Cancer Research, National Cancer Institute, National Institutes of Health, Bethesda, MD; ^2^ Houston Methodist Research Institute, Houston, TX

**Keywords:** cSrc signaling, phosphorylation of thyroid hormone receptor β1, tumor suppressor, breast cancer cells, xenograft models

## Abstract

Association studies suggest that the thyroid hormone receptor β1 (TRβ1) could function as a tumor suppressor in cancer cells. However, the underlying molecular mechanisms remain to be elucidated. We explored how TRβ1 acted as a tumor suppressor in breast cancer MDA cells. Proliferation and invasiveness were markedly inhibited in cells stably expressing TRβ1 (MDA-TRβ1 cells). cSrc-phosphorylated TRβ1 at Y406 signaled T3-induced degradation. Mutation of Y406 to Phe (TRβ1Y406F) did not affect T3 binding affinity, but blocked T3-induced degradation in cells. Importantly, cell-based studies showed that TRβ1Y406F lost the inhibitory effects by TRβ1 on cell proliferation and invasion. Consistently, in xenograft models, MDA-TRβ1 cells exhibited significantly slower tumor growth rates than those of Neo control cells. In contrast, the tumor growth rates of MDA-TRβ1Y406F cells were indistinguishable from those of Neo control cells. We further showed that markedly more TRβ1Y406F than TRβ1 was physically associated with cSrc in cells, leading to constitutive activation of cSrc-FAK-ERK signaling. In contrast, degradation of T3-bound TRβ1 complexed with cSrc attenuated signaling to decrease cell proliferation and invasiveness, thus confirming TRβ1 as a tumor suppressor. Thus, the present studies suggested that TRβ1 could be tested as a novel potential therapeutic target.

## INTRODUCTION

Thyroid hormone receptors (TRs) are ligand-dependent transcription factors that mediate the biological activities of the thyroid hormone T3. The TR isoforms—,α1, β1 and β2—are encoded by the *THRA* and *THRB* genes, respectively, located on two different chromosomes. These TR isoforms share extensive sequence homology in the DNA and T3 binding domains, but differ in the amino terminal A/B domains [1]. TR binds to the thyroid hormone response elements (TREs) and recruits nuclear co-regulatory proteins to regulate gene transcription. In the absence of T3, TRs recruit the nuclear corepressors for transcriptional repression on the T3-positively-regulated genes. In the presence of T3, the T3-bound TR undergoes structural changes that result in the release of co-repressors, thus allowing recruitment of nuclear receptor coactivators to facilitate transcription activation [2, 3]. Recent studies also suggest that TRβ1 could act via protein-protein interaction with the PI3K-regulatory subunit p85α in extra-nuclear sites to initiate intracellular signaling [4-6].

There has been recent progress in understanding the molecular mechanisms by which TR functions to mediate T3 biological activities in normal growth, differentiation, and development, but the roles of TRs in human cancers are less well understood. Early studies indicated that truncations and/or deletions of chromosome 3p where the *THRB* gene is located are closely associated with human malignancies including lung, melanoma, breast, head and neck, renal cell, uterine cervical, ovarian, and testicular tumors [7-12]. Moreover, decreased expression due to silencing of the *THRB* gene by promoter hypermethylation has been found in human cancer including breast, lung, and thyroid carcinoma [13-16]. These association studies raised the possibility that TRs could function as tumor suppressors in human cancers.

Recent studies have presented compelling evidence to support the notion that TRβ1 could function as a tumor suppressor. The expression of TRβ1 in hepatocarcinoma and breast cancer cells reduces tumor growth, causes partial mesenchymal-to-epithelial cell transition, and has a striking inhibitory effect on invasiveness, extravasation, and metastasis formation in mice [17]. Moreover, in neuroblastoma cells stably expressing TRβ1, the transcriptional response mediated by the Ras/mitogen-activated protein kinase/ribosomal-S6 subunit kinase-signaling pathway is inhibited. Moreover, fibroblast transformation and tumor formation in nude mice induced by oncogenic *ras* are blocked when TRβ1 is expressed [18]. The tumor suppressor function of TRβ1 was also demonstrated in human follicular thyroid cancer (FTC) cells. Expression of TRβ in FTC-133 cells reduces cancer cell proliferation and impedes migration of tumor cells through inhibition of the AKT-mTOR-p70 S6K pathway. TRβ1 expression in FTC cells inhibits tumor growth in xenograft models [19].

Despite growing evidence that TRβ1 is a tumor suppressor, the molecular mechanisms have yet to be fully elucidated. Our previous studies suggested that TRβ1 could initiate its actions via extra-nuclear sites [4, 5, 20]. Based on these findings, we hypothesized that extra-nuclear TRβ1 signaling could be mediated by phosphorylation cascades. Accordingly, we stably expressed TRβ1 in breast cancer MDA cells and found that proliferation and invasiveness were markedly inhibited in cells stably expressing TRβ1 (MDA-TRβ1 cells). Biochemical analyses showed that TRβ1 was phosphorylated by Src kinase at Y406. Further molecular studies demonstrated that phosphorylation by cSrc at TRβ1Y406 signaled T3-induced degradation, thereby markedly attenuating cSrc signaling to suppress cell proliferation and invasiveness. When TRβ1Y406 was mutated to Phe (TR1Y406F), no T3-induced degradation occurred, resulting in constitutive activation of cSrc signaling to promote oncogenesis. The present studies uncovered a novel mechanism by which TRβ1 could function as a tumor suppressor via cSrc-dependent phosphorylation.

## RESULTS

### TRβ1 is phosphorylated at tyrosine406 (Y406) by cSrc kinase

We have recently shown that TRβ1 acts as a tumor suppressor in human thyroid Tori cells (HTori). HTori cells were derived from transfection of human primary thyroid follicular epithelial cells with a plasmid containing an origin-defective SV40 genome (SVori-) [21]. We showed that one mechanism by which TRβ1 acts as a tumor suppressor in HTori cells is by physical interaction with SV40Tag. This leads to inactivation of the oncogenic actions of SV40Tag by blocking its recruitment of the retinoblastoma protein (Rb) and p53 tumor suppressors [22]. In view of the critical roles of phosphorylation in cellular functions, and the recent findings that TRβ1 could act via extra-nuclear sites [4, 5, 20] we hypothesized that the tumor suppressor activity of TRβ1 could involve phosphorylation. We therefore tested this possibility by determining whether TRβ1 was phosphorylated at the tyrosine residues (Tyr). We first used anti-Tyr antibodies to immunoprecipitate cellular proteins phosphorylated at Tyr; this step was followed by Western blot analysis using anti-TRβ1 antibodies.

**Figure 1:**
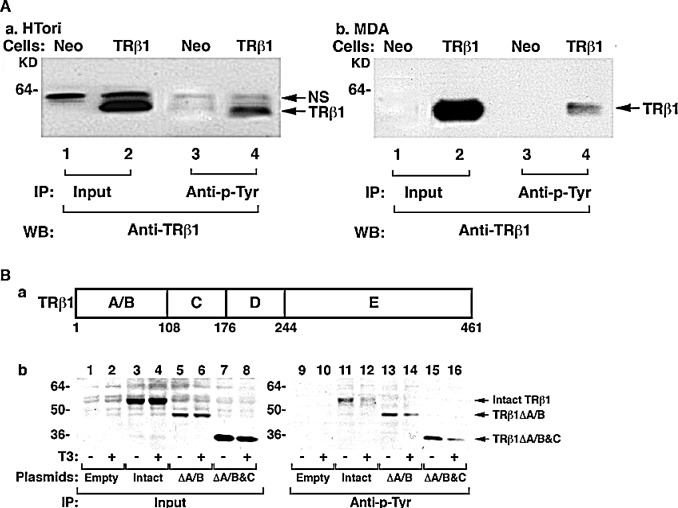
TRβ1 is phosphorylated at Tyr in human HTori and MDA breast cancer cells (A) HTori-TRβ1 (a) and MDA-TRβ1 cells (b) together with Neo control cells were immunoprecipitated with p-Tyr antibodies followed by Western blot analysis for detecting TRβ1 was described Materials and Methods. Lanes are as marked. (B) Mapping of the domains of TRβ1 that contained the phosphorylated Tyr sites. (a) Schematic representation of the TRβ1 with domain boundaries marked with amino acid numbers. (b) Expression plasmids for full-length TRβ1, ΔA/B TRβ1, ΔA/B/C TRβ1 were transiently transfected into MDA cells, followed by treatment without or with T3 briefly for 15 min. Cellular extracts were prepared and immunoprecipitated with anti-Tyr antibodies followed by Western blot analysis using anti-TRβ1, J53, recognizing the “D+E” domains of TRβ1. Lanes 1-8 are the input and lanes 9-16 are the corresponding truncated proteins detected as marked.

In HTori cells stably expressing TRβ1 (HTori-TRβ1 cells; Figure 1A-a), a strong specific Tyr phosphorylated-TRβ1 was detected (lane 4), whereas in the control Neo cells, no specific band was observed (lane 3). The corresponding input amounts are shown in Lanes 1 and 2 (Figure 1A-a). These results indicated that TRβ1 in HTori-TRβ1 cells was phosphorylated at Tyr residues. That TRβ1 was also phosphorylated at Tyr residues in other cancer cells was shown in MDA-MB-468 cells stably expressing TRβ1 (MDA-TRβ1 cells). A specific Tyr phosphorylated-TRβ1 was detected in MDA cells stably expressing TRβ1 (lane 4; Figure 1A-b), but not in the corresponding Neo control cells (lane 3; Figure 1A-b). Similarly, TRβ1 phosphorylated at Tyr was also found in another breast cancer cell line, MCF-7 cells (data not shown). These results indicate that TRβ1 phosphorylated at Tyr was not limited to HTori (thyroid) cells, but also in breast cancer cells. We therefore further sought to identify the site(s) of Tyr phosphorylation and its role in TRβ1 functioning as a tumor suppressor in MDA cells.

To locate the domains of TRβ1 in which Tyr was phosphorylated, we transfected intact and truncated TRβ1 expression plasmids into MDA cells (Figure 1B-a). Next we performed Western blot analysis with anti-C-terminal TR antibody after immunoprecipitation with anti-Tyr antibody. Figure 1B-b shows that phosphorylation at the Tyr was detected in the intact TRβ1 (lanes 11 and 12), truncated TRβ1 lacking the A/B domain (ΔA/B) (lanes 13 and 14), and truncated TRβ1 lacking the A/B & C domains (ΔA/B&C; lanes 15 and 16). These results indicate that the Tyr phosphorylation sites were located in the ligand binding domain (LBD, domains D&E, amino acid position 176-461; see Figure 1B-a). It is important to note that in the presence of T3, a lower TRβ1 amount (lane 12), ΔA/B (lane 14), and ΔA/B&C (lane 16) were found, respectively, than in the absence of T3 (lanes 11, 13, and 15). These T3-induced decreases were not caused by different input amounts as equal amounts were used (lanes 3&4, 5&6, and 7&8, Figure 1B). These results indicate that Tyr phosphorylation of the liganded TRβ1 decreased the stability of TRβ1.

**Figure 2:**
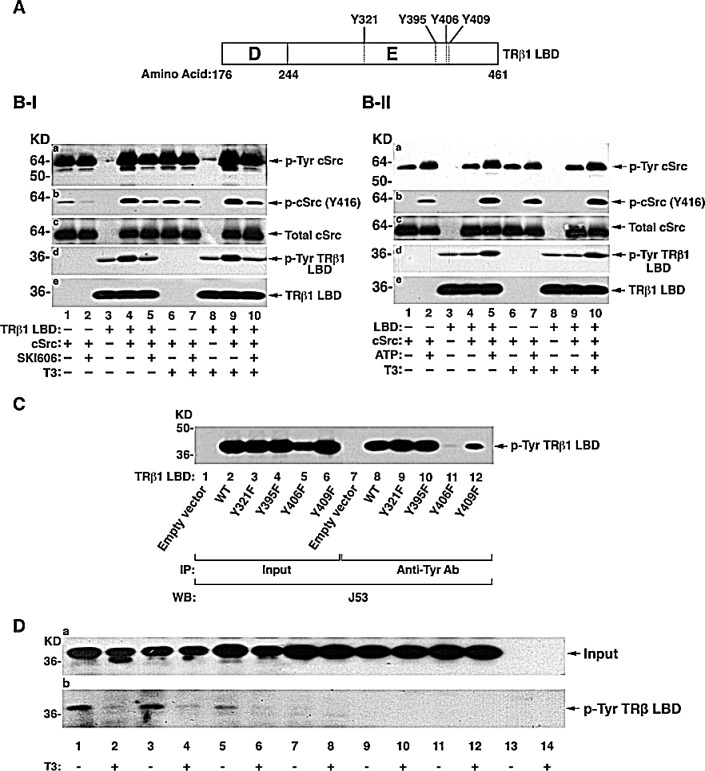
Identification of the TRβ1 domain containing cSrc phosphorylated tyrosine site (A) Schematic representation of ligand binding domain (LBD) of TRβ1. The D and E domain boundaries are marked. The four tyrosine residues in the domain E are indicated. (B-I) Effect of cSrc inhibitor, SKI606, on the *in vitro* phosphorylation of purified LBD with cSrc in the absence of T3 (lanes 1-5) or presence of T3 (lanes 6-10) and other conditions as marked. (B-II) Effect of ATP on the *in vitro* phosphorylation of purified LBD with cSrc in the absence of T3 (lanes 1-5) or presence of T3 (lanes 6-10) and other conditions as marked. (C) MDA cells were transfected with expression vectors for LBD with mutation at Y321F, Y395F, Y406F or Y409F. Cellular extracts were immunoprecipitated with anti-Tyr antibodies followed by anti-TRβ1, J53 as described in Figure 1B. (D). Src mediated tyrosine phosphorylation of TRβ1 Y406F inhibited by SKI606. MDA cells were transfected with expression vectors for TRβ1LBD or LBDY406F with/ or without SKI606 (0.5 μM or 1 μM for 8 hours) and with or without T3 (100 nM for 15 min). Cellular extracts were immunoprecipitated with anti-Tyr antibodies followed by anti-TRβ1, J53. Lanes are as marked.

The LBD of TRβ1 has four potential Tyr residues (Y321, Y395, Y406, and Y409) that could be phosphorylated by tyrosine kinases (Figure 2A). To identify the tyrosine kinases that could phosphorylate TRβ1, we used the purified LBD and screened for tyrosine kinases using *in vitro* kinase systems. cSrc was one of the few purified tyrosine kinases available for testing. Since it is known that cSrc is self-phosphorylated [23], we first confirmed that the purified cSrc was active by Western blotting using anti-Tyr antibodies in the presence or absence of T3 (Figure 2B-I). Phosphorylated cSrc was detected in the absence (lanes 1 & 2) or presence of LBD (lanes 4 & 5). The cSrc self-phosphorylation was not affected by T3 (lanes 1-5 vs 6-10). Using cSrc specific phosphorylation site anti-Y416 antibodies, the activity of cSrc was further confirmed as cSrc phosphorylation at Y416 was inhibited by SKI606 (compare lanes 2 with 1; 5 to 4; panel b, Figure 2B-Ib). As a control, panel c (Figure 2B-Ic) shows that total cSrc was not affected by the presence of cSrc inhibitor, or T3). Moreover, in the absence of ATP, the extent of phosphorylation at Y416 was inhibited (compare lanes 1 to 2, 4 to 5, 6 to 7 and 9 to 10, Figure 2B-II, panel b) without changing the total cSrc protein levels (Figure 2B-II, panel c). These data show that the purified c-Src was active in the *in vitro* kinase system. Panel d in Figure 2B-I shows that TRβ1 LBD was phosphorylated at the Tyr residues in the absence of T3 (lane 4) by purified cSrc kinase. In the presence of cSrc specific inhibitor, SKI606, the extent of phosphorylation was inhibited to the basal level (lane 5 vs lane 3). In the presence of T3, TRβ1 LBD was similarly phosphorylated (lane 9) and it was also inhibited by cSrc inhibitor (lane 10). Moreover, in the absence of ATP, no cSrc-dependent phosphorylation was observed (compare lanes 4 to 5 and lane 9 to 10, Figure 2B-II-d). Taken together, these data indicate that TRβ1 LBD was phosphorylated by cSrc and that the phosphorylation of Tyr was T3 independent in the *in vitro* kinase system.

To identify which Tyr residues in the TRβ1 LBD were phosphorylated by cSrc in cells, we constructed expression plasmids in which each of the four Tyr residues in the LBD was mutated from Tyr to phenylalanine (Phe). After these plasmids were transfected into MDA cells followed by immunoprecipitation and Western blot analysis (Figure 2C), Y406F (see Figure 2A) was found not phosphorylated in cells (lane 11, Figure 2C), while mutations in Y321, Y395, and Y409 were phosphorylated to a similar extent as that in WT LBD (lanes 9, 10, 12 vs 8). These results indicate that Y406 was the Tyr residue in the LBD phosphorylated by cSrc kinase in MDA cells.

We next evaluated whether cSrc indeed was the cellular kinase that phosphorylated Y406. We therefore treated cells stably expressing WT TRβ1LBD or Y406F in the absence or increasing concentrations (0.5 and 1μM) of cSRC-specific inhibitor, SKI606. Figure 2D-a shows that Tyr phosphorylation of WT TRβ1LBD was nearly completely inhibited in in the presence of SKI606 at 1 μM (lanes 5 and 6 vs lane 1 and 2, respectively) in the absence or presence of T3, whereas, no Tyr phosphorylation of LBDY406F was detected at any conditions (lanes 7-12). These results further confirmed that Y406 was the phosphorylation site and that in cells cSrc was the cellular kinase that phosphorylated the TRβ1LBD.

**Figure 3:**
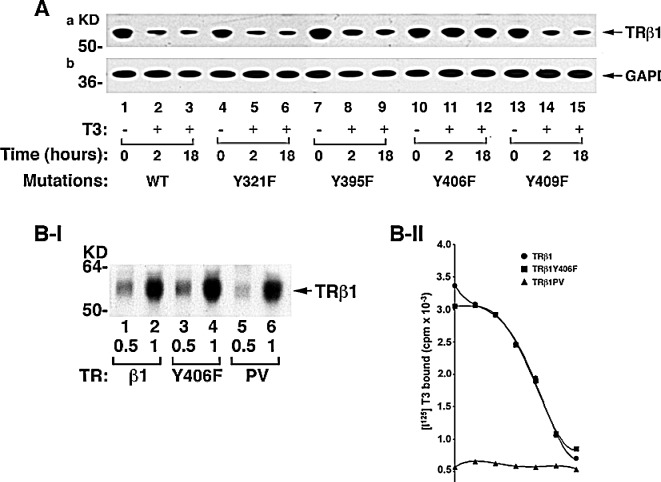
Identification of the cSrc-targeted phosphorylated Y406 in TRβ1 (A) TRβ1 phosphorylated at Y406 by cSrc resistant to T3-induced degradation. MDA cells were transfected with expression vectors for intact TRβ1 with mutations at Y321F, Y395F, Y406F, or Y409F. Cell lysates were prepared after treatment of cells without or with T3 for 8 and 18 hours. (B) Mutation of Y406 to Phe did not affect the T3 binding activity. I. The amounts of *in vitro* translated TRs used in the T3 binding assays were quantified by western blot using anti-TRβ1 antibody. II. An equal amount of TRβ1 (●), TRβ1Y406F (■), and TRβ1PV (▲) were used in the competitive T3 binding assay as described in Materials and Methods.

We further constructed expression plasmids in which each of the four Tyr residues in intact TRβ1 was mutated from Tyr to Phe and expressed them in MDA cells. Finding that the phosphorylated LBD was degraded in the presence of T3 (Figure 1B) prompted us to examine the effect of the loss of Tyr phosphorylation on the stability of T3-bound intact TRβ1. Figure 3A shows that WT TRβ1 was nearly completely degraded after 18 hours’ treatment of cells with T3 (lanes 3 vs 1). A similar extent of degradation was found for TRβ1Y321F (lanes 6 vs 4), TRβ1Y395F (lanes 9 vs 7), and TRβ1Y409F (lanes 15 vs 13). However, no T3-induced degradation was observed for TRβ1Y406F (lanes 10-12). These results indicate that phosphorylation at TRβ1Y406 was critical for T3-induced degradation. That TRβ1Y406F failed to be degraded in the presence of T3 could be caused by the loss of T3 binding activity. We therefore compared the T3 binding avidity of WT TRβ1 and TRβ1Y406F. We also included TRβ1PV (PV) that has a mutation at the C-terminal 14 amino acids and has completely lost T3 binding activity as a control [24]. Figure 3B-I shows that equal amounts of TRβ1 (lanes 1-2), TRβ1Y406F (lanes 3-4), and PV (lanes 5-6) prepared by *in vitro* transcription/translation were used in the binding assays. Figure 3B-II shows no binding was detected for PV, as expected. Interestingly, no differences in the competitive displacement curves were found between TRβ1 WT and mutant TRβ1Y406F. These binding data indicate that mutation from Tyr to Phe at residue 406 did not affect T3 binding to TRβ1. Thus, phosphorylation at Y406 by cSrc kinase was critical for signaling the degradation of TRβ1.

### TRβ1Y406F loses the tumor suppressor activities of TRβ1

**Figure 4:**
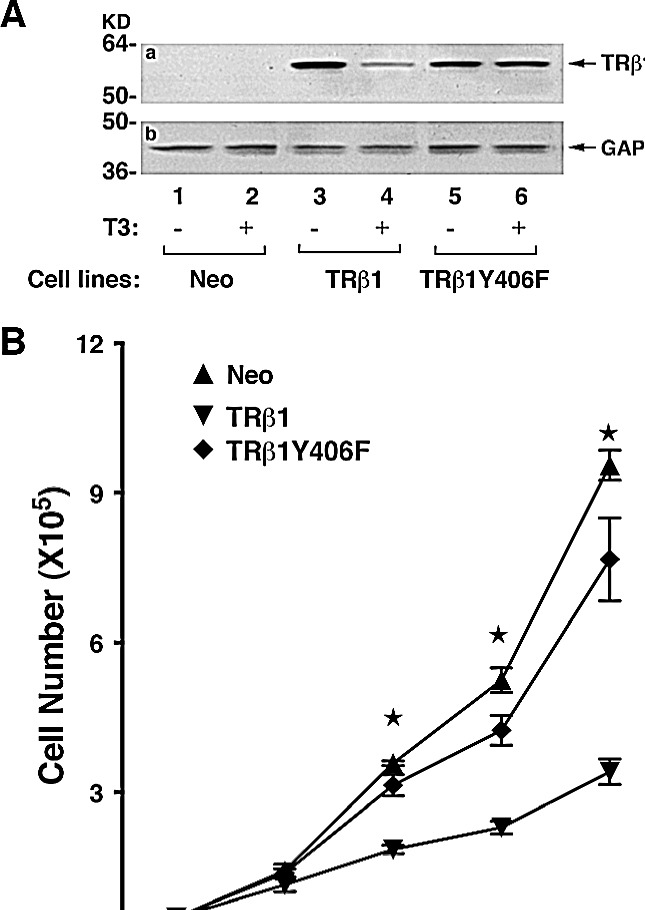
Comparison of growth rates of MDA-TRβ1, MDA-TRβ1Y406F and Neo control cells (A) TRβ1 and TRβ1Y406F protein abundance in of MDA-TRβ1 (lanes 3-4) and MDA-TRβ1Y406F cells (lanes 5-6). T3 induced degradation of TRβ1 (lane 4), but not TRβ1Y406F protein (lane 6), in the presence T3 (lanes 4 and 6), but not without T3 (lane 3 and 5). Lanes 1 & 2 are from the control Neo cells. (B) Cell growth was analyzed as described in Materials and Methods. Data are expressed as mean ± standard error (SE) (n=3) and analyzed by one-way ANOVA with Tukey’s post-hoc test, “a”; p<0.05. Cell lines are as marked.

To elucidate the functional consequences due to the loss of phosphorylation at Y406, we prepared MDA cell lines stably expressing TRβ1Y406F (MDA-TRβ1Y406F cells). Figure 4A shows the representative clone expressing TRβ1Y406F that was unable to undergo T3-induced degradation (lanes 6 vs 5), whereas the WT TRβ1 protein level was degraded in the presence of T3 (lane 4 vs 3). We have recently shown that TRβ1 stably expressed in human thyroid cancer cells, FTC-133 [19], and MCF-7 breast cancer cells [25] functions as a tumor suppressor by inhibiting cell proliferation. We therefore evaluated whether the loss of phosphorylation at Y406 could affect the tumor suppressor functions of WT TRβ1. Figure 4B shows that the proliferation rate of MDA cells stably expressing TRβ1 (MDA-TRβ1 cells) was significantly lower than that of the control Neo cells. These findings are consistent with those found in human FTC-133 and MCF-7 cells [19, 25]. Remarkably, MDA-TRβ1Y406F cells exhibited proliferation rates indistinguishable from those of the Neo control cells (Figure 4B). These results indicate that inability to be phosphorylated at Tyr406 led to the loss of inhibitory effects in cell proliferation by WT TRβ1.

**Figure 5:**
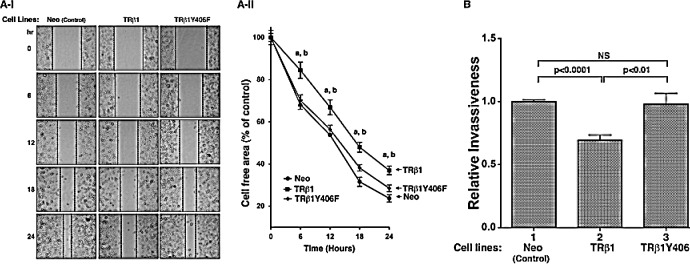
Comparison of cell migration (A) and invasiveness (B) of MDA-TRβ1, MDA-TRβ1Y406F and Neo control cells (A-I) Representative pictures of cell wound healing in Neo cells, MDA-TRβ1 cells, and MDA-TRβ1Y406F cells at 0, 6, 12, 18 and 24 hours. (A-II) Cell migration rates determined from results observed in (A-I). Data are expressed as mean ± standard error (SE) (n=3), a; p<0.05 MDA-TRβ1 cells vs Neo control cells, b; p<0.05 MDA-TRβ1 cells vs MDA-TRβ1Y406F cells. (B) Comparison of invasiveness of MDA-TRβ1 cells, MDA-TRβ1Y406F and Neo control cells was carried out as described in Materials and Methods. Data are presented as mean ± SE (n=3) and analyzed by one-way ANOVA with Tukey’s post-hoc test.

We have also shown previously that WT TRβ1 acted to inhibit cell migration in FTC-133 cells [19]. We therefore further assessed the impact of the loss of phosphorylation at Y406 by cell migration and invasion. Figure 5A-I shows that TRβ1 stably expressed in MDA cells impeded cell migration as compared with the control Neo cells. TRβ1Y406F stably expressed in MDA cells lost the inhibitory effects as these cells migrated at a rate similar to that in the control Neo cells. The migration distances from three different cell lines were measured and compared quantitatively as shown in Figure 5A-II. Results indicated that TRβ1 stably expressed in MDA cells impeded cell migration. In contrast, MDA-TRβ1Y406F cells had a migration rate similar to that in the Neo control cells. Moreover, we also evaluated the invasiveness of MDA-TRβ1Y406F cells. Figure 5B shows that while MDA-TRβ1 cells had a 30% lower invasiveness than the control Neo cells (compare bars 2 with bar 1), MDA-TRβ1Y406F cells had similar extent of invasiveness as the control Neo cells (compare bars 3 with 1). Taken together, these data indicate that the inability to be phosphorylated at Y406 led to loss of the tumor suppressor activity of WT TRβ1.

**Figure 6:**
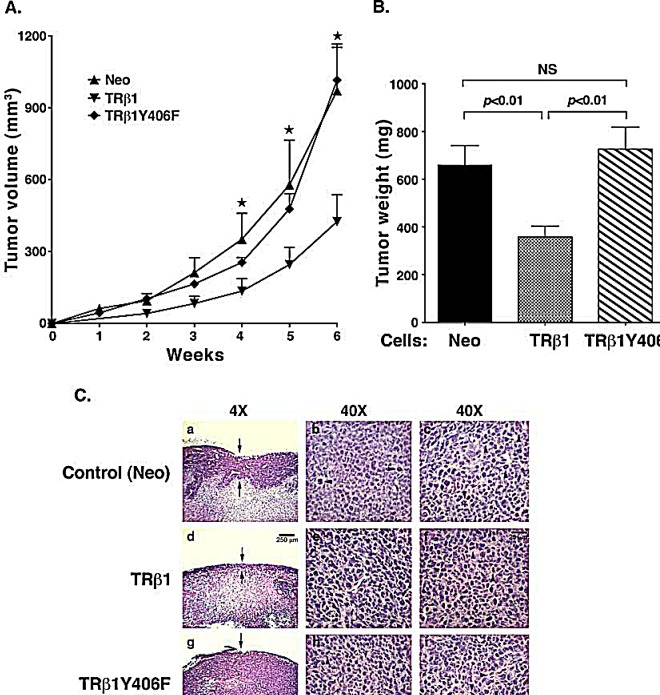
Comparison of tumor growth rates of MDA-TRβ1, MDA-TRβ1Y406F, and Neo control cells (A) Equal numbers of cells for 3 cell lines were inoculated onto the right flank of mice 6-week-old female athymic NCr-nu/nu mice. Tumor sizes were measured weekly and the rates of tumor growth were compared. (B) Tumors were dissected at the endpoint and the weight was determined. The data are expressed as mean ± SE (n=6), ⋆; p<0.05 MDA-TRβ1vs Neo cells or MDA-TRβ1Y406F. (C). Comparison of histological characteristics in tumors derived from Neo cells (panels a, b and c), MDA-TRβ1 (panels d, e and f) and from MDA-TRβ1Y406F cells (panels g, h, i). The magnification was 4X in panel a, d and g to indicate increased thickness of the viable areas in tumors derived from MDA-TRβ1Y406F cells (panel g). The magnification was 40X in b & c (two tumors derived from Neo cells), e & f (two tumors derived from MDA-TRβ1) and h & I (two tumors derived from MDA-TRβ1Y406F cells).

We further confirmed the cell-based findings by *in vivo* studies using xenograft models. We injected MDA-TRβ1Y406F cells, MDA-TRβ1 cells, or Neo controls into athymic NCr-*nu/nu* mice. As shown in Figure 6A-I, the tumor growth rate for MDA-TRβ1 cells was clearly significantly slower than that of Neo control cells, indicating TRβ1 acted as a tumor suppressor *in vivo*. In contrast, the tumor growth rate from MDA-TRβ1Y406F cells was indistinguishable from that of Neo control cells. A quantitative comparison of tumor weights derived from the three cell lines is shown in Figure 6B. The results indicated that there was no significant differences in tumor weights between Neo and MDA-TRβ1Y406F cells, but the tumor weight from MDA-TRβ1 cells was ~50% lower than that of Neo and MDA-TRβ1Y406F cells. Moreover, we examined the histological characteristics of the H & E-stained tumor sections derived from the three cell lines (Figure 6C). The 40X images show the viable areas in each tumor (each from two tumors) and the arrows point to mitotic figures indicating cell replication. The striking difference was apparent in the thickness of the viable tumor areas at the edges, since all the tumors have some central necrosis. This is shown in the 4X images with the arrows indicating the thickness of the viable areas (Figure 6C, panels a, d and g for the control Neo cell, MDA-TRβ1 cells and MDA-TRβ1Y406F cells, respectively). Note that the tumors derived from MDA-TRβ1 cells had less viable tumor area at the edge and the tumor derived from MDA-TRβ1Y406F cells had the thickest viable areas, consistent with its greater growth. Take together, these results support the cell-based studies that the phosphorylation at Y406 of TRβ1 by cSrc was critical for tumor suppressor functions of TRβ1. The inability for Y406 to be phosphorylated by cSrc kinase led to the loss of tumor suppressor activities of wild-type TRβ1.

### TRβ1Y406F loses the inhibitory effects on activated cSrc signaling by TRβ1

**Figure 7:**
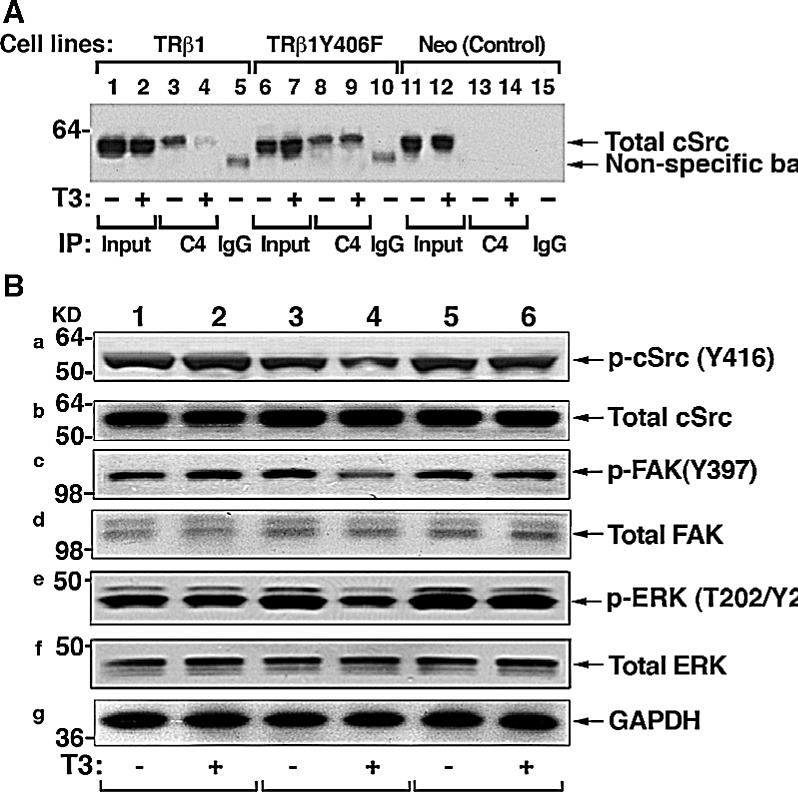
cSrc signal pathway is constitutively activated by TR1Y406F via stable protein-protein interaction (A) Comparison of co-immunoprecipitated Src-TRβ1 (lanes 3 & 4) and cSrc-TRβ1Y406F (8 & 9) in the absence (lanes 3&8) or presence (lanes 4 & 9). No co-immunoprecipitated bands were detected in the Neo control cells (lanes 13 & 14). Lanes 1,2, 6, 7, 11, and 12 show the corresponding input. The IgG controls are shown in lanes 5, 10 and 15. co-immunoprecipitation was carried out as described in Materials and Methods. (B) Constitutive activation of c-Src signal pathway in MDA-TRβ1Y406F cells. Western blot analysis of p-Src (Y416), total Src, p-FAK (Y397), total FAK, p-ERK (T202/Y204), total ERK, and GAPDH (as loading control) in Neo cells (lanes 1 & 2), MDA-TRβ1Y cells, and MDA-TRβ1Y406F cells in the absence (lanes 1,3, and 5) or presence of T3 (lanes 2, 4, and 6) was carried out as described in Materials and Methods.

**Figure 8:**
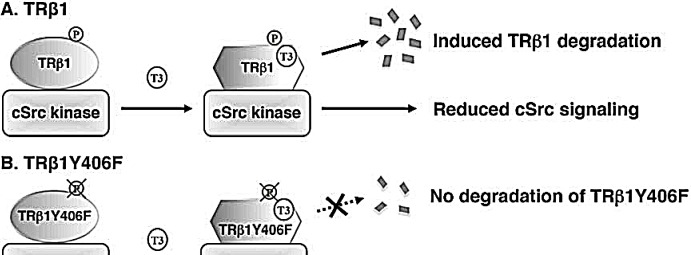
A proposed molecular model for the effects of tyrosine phosphorylation of TRβ1 in the degradation of TRβ1 and regulation of cSrc signaling (A) Phosphorylation of Y406 triggers the T3-induced degradation of TRβ1, releasing TRβ1 from complexing with cSrc and attenuating cSrc signaling. (B) TRβ1Y406F, unable to be phosphorylated by cSrc, thereby remaining stably associated with cSrc, leading to constitutive activation of cSrc signaling to increase cell proliferation and invasion.

That TRβ1 was phosphorylated by cSrc at Tyr406 prompted us to investigate how the loss of phosphorylation affected the association of TRβ1 with cSrc by co-immunoprecipitation assay. Using total cellular extracts, we showed that TRβ1 interacted with cSrc, but in the presence of T3, markedly less TRβ1 was associated with cSrc in the presence of T3 (compare lane 4 with lane 3). In contrast, a similar extent of association of TRβ1Y406F with cSrc was detected whether T3 was present or not (lanes 8 & 9). Lanes 13-15 of Figure 7A were the controls using Neo cells in which no TRβ1 was present. These results indicate that upon phosphorylation, T3 induced the degradation of TRβ1, freeing cSrc from association with TRβ1. In contrast, T3 did not induce degradation of TRβ1Y406F, indicating it is constitutively associated with cSrc (see also the molecular model in Figure 8).

Since it is known that cSrc can be activated via protein-protein interaction with receptors and activators [26, 27], we therefore ascertained whether constitutive association of cSrc with TRβ1Y406F affected the activity of cSrc. Consistent with T3-induced degradation of TRβ1, less p-cSrc (Y416) was detected in MDA-TRβ1 cells (lane 4 vs lane 3, Figure 7B-a), but similar levels of p-cSrc as that in the control Neo cells (lane 1 & 2; Figure 7B-a) were detected in MDA-TRβ1Y406F cells (lanes 5 & 6, Figure 7B-a). The total cSrc levels remained similar in the three cell lines whether T3 was present or not (Figure 7B-b). That cSrc was constitutively activated in MDA-TRβ1Y406F cells prompted us to probe its downstream effectors and signaling targets. Focal adhesion kinase (FAK), upon phosphorylation at Y397, provides a binding site for cSrc, relaying the activated signals to RAS (e.g., KRAS), triggering signaling cascades to ERK activation [28]. Indeed, we found that p-Y397 was lower in MDA-TRβ1 cells than in Neo cells and MDA-TRβ1Y406F cells in the presence of T3 (compare lane 4 with lanes 2, & 6), while similar levels of p-Y397 were detected in MDA-TRβ1Y406F cells whether T3 was present or not (lane 5 & 6, panel c). The downstream effector, p-ERK, was lower in cells stably expressing TRβ1 (lane 4, panel e, Figure 7B) than in Neo cells (lanes 1 & 2), but remained elevated in MDA-TRβ1Y406F cells (panel e, lanes 5 & 6). Total ERK, however, remained unaffected (panel f). Panel g shows the protein levels of GAPDH as loading controls. These results indicate that phosphorylation of TRβ1 at Y406 triggered the T3-induced degradation that weakened the activated state of cSrc. As a result, cSrc-FAK-ERK signaling was attenuated, thereby suppressing the oncogenic events. In contrast, inability of TRβ1Y406F to be phosphorylated by cSrc kinase, remained associated with the activated cSrc. The inability of TRβ1Y406F to undergo T3-induced degradation led to the loss of tumor suppressor activity by TRβ1.

## DISCUSSION

While evidence has been accumulating to indicate that TRβ1 could function as a tumor suppressor [17-19], how TRβ1 acts to exert such an important function is just beginning to be unraveled. The present studies identified one mechanism by which TRβ1 could function as a tumor suppressor via cSrc-dependent phosphorylation. Our cell-based studies showed activation of cSrc when complexed with the unliganded TRβ1, resulting in downstream signaling to increase cell proliferation and invasion. However, phosphorylation by cSrc at Y406 signaled degradation of TRβ in the presence of T3. Degradation of T3-bound TRβ1 freed cSrc from TRβ-associated complex, resulting in the attenuation of cSrc-FAK-ERK signaling (Figure 8A). This molecular model was further strengthened by mutational analysis in that mutation of Y406 to Phe led to a mutant that was constitutively associated with cSrc. TRβ1Y406F bound to T3 with similar binding affinity as wild-type TRβ1. TRβ1Y406F cannot be phosphorylated by cSrc, thus lacking the signal to trigger degradation of T3-bound TRβ1 (Figure 8B). As a result, cSrc was constitutively associated with TRβY406F, resulting in a sustained activated cSrc-FAK-KRAS-ERK signaling. Thus, the present studies uncovered a novel molecular mechanism by which TRβ1 could function as a tumor suppressor via protein-protein interaction with cSrc kinase.

However, at present, it is not clear whether the mechanism shown in Figure 8 for T3-bound TRβ1 could be extended to the liganded TRα1. Furuya et al found that the liganded TRα1 could stimulate proliferation of pancreatic β-cells in adult animals [29]. While we had previously shown that in CV1 cells, the transfected TRα1 could also undergo T3-dependent proteasomal degradation similarly as shown for T3-bound TRβ1 [30], it is currently unknown whether the T3-dependent proteasomal degradation of the liganded TRα1 could also occur in pancreatic β-cells. Moreover, it is also unknown whether TRα1 could be similarly phosphorylated by cSrc at the tyrosine residues as in TRβ1. These questions would need to be studied in the future.

Post-translational modification of steroid hormone nuclear receptors by phosphorylation is well studied. Evidence has been presented to indicate that phosphorylation affects the functions of steroid hormone nuclear receptors not only in normal physiology, but also in pathophysiology of many diseases including cancers, diabetes, and obesity, among others [31]. For example, the progesterone receptor (PR) and estrogen receptors (ER) are phosphorylated by multiple protein kinases either basally or in response to ligand binding. Phosphorylation leads to increased transcriptional activity of these receptors to drive the expression of target genes important for cell cycle progression, proliferation, and survival [32]. Alterations in the expression of these key regulators could impact breast cancer progression. Phosphorylation of PR and ER is also involved in nongenomic membrane-associated actions [32, 33]. In contrast to steroid hormone receptors, however, how phosphorylation affects the functions of TRs is less studied. Early reports have shown that phosphorylation stimulates the transcriptional activity of TRβ1 over-expressed in COS1 cells [34]. While the phosphorylation residues from TRβ1 over-expressed in COS1 cells were shown to be serine, threonine, and tyrosine in the ratios of 8.5:1:0.5, the sites of these resides have not been identified, nor was the identity of kinases responsible for phosphorylation elucidated. Therefore it was not possible to elucidate the functions of the site-dependent phosphorylation. The present study identified Y406 as the site phosphorylated by cSrc kinase. It served as a signal for T3-induced degradation of TRβ1, which in turn affected the activity of cSrc in intracellular cSrc-FAK-ERK signaling pathways. That the activity of TRβ1 was modulated by complexing with cSrc in a T3-depedent manner is not without precedent. It is of interest to point out that treatment of breast cancer cells with progestin activates the MAPK signaling that requires PR-cSrc interaction [35]. Complexing of ER with cSrc and scaffold proteins was also found to stimulate cSrc activity by increasing downstream signaling pathways including PI3K and MAPK [33, 36]. ERα is phosphorylated at Y537 by cSrc *in vivo*, and this phosphorylation is required for triggering DNA synthesis and tumor growth [37]. Moreover, an ER activity stimulator, hexachlorobenzene (an endocrine disrupter), enhances ERαY537 phosphorylation and ERα-cSrc physical interaction [38]. Therefore TRβ1, ER, and PR could exert cSrc-dependent cellular functions via direct protein-protein interaction.

However, the functional outcomes of the cSrc-dependent TRβ1 action differed from that of PR and ER in that T3-bound TRβ1 was triggered by cSrc-dependent phosphorylation to undergo degradation, resulting in the attenuation of cSrc-FAK-ERK signaling. In contrast, the cSrc-dependent ER and PR actions led to stimulated downstream signaling pathways including PI3K and MAPK. In breast cancer as well as in other cancers such as small cell lung, head and neck, renal cell, uterine cervical, and ovarian cancers, the expression of the *THRB* gene is frequently silenced either via chromosomal truncation and/or deletion in chromosome 3p where *THRB* lies, or via epigenetic changes in the promoter region of the *THRB* gene (e.g., hypermethylation; [10]. In light of these findings, one could speculate that cancer cells may have evolved the above mechanisms to silence the expression of the *THRB* gene to evade the tumor suppressor actions of TRβ1 in growth, proliferation, and invasion. This notion would suggest that reactivation of the *THRB* gene in cancer cells would lead to attenuation of cancer phenotypes. Indeed, this hypothesis was recently tested and validated in human FTC-236 cells [39]. Evaluation of promoter methylation and the expression of the *THRB* gene in tissue specimens from patients with differentiated thyroid carcinoma and in several human thyroid cancer cell lines (e.g., FTC-236) showed a positive correlation between the extent of promoter hypermethylation of the *THRB* gene and the progression of differentiated thyroid carcinoma. When FTC-236 cells were treated with demethylation agents such as 5′aza-CdR and zebularine, the expression of the *THRB* gene was reactivated concurrently with inhibition of cancer cell proliferation, migration, and tumor growth in xenograft models [39]. It is known that cSrc is aberrantly activated in many cancers [40, 41]. That TRβ1 could down regulate the activity of cSrc in the presence of T3 via phosphorylation at Y406 would suggest that TRβ1 could be tested as a potential novel therapeutic target.

## MATERIALS AND METHODS

### Cell lines

HTori cells were a generous gift from Yuri Nikiforov of the University of Pittsburgh Medical Center, Pittsburgh, Pennsylvania. Establishment of HTori cells stably expressing either TRβ1 or the control gene (Neo) was described previously [42]. MDA-MB-468 cells were from Ana Aranda (Universidad Auto’noma de Madrid, Madrid, Spain).

### *In vitro* kinase reaction with cSrc kinase

One μg of purified human TRβ1 ligand binding domain [43] was incubated with 50 ng Src kinase (Millipore cat. 14-326) in *in vitro* kinase buffer containing 60 mM HEPES (pH7.5), 5 mM MgCl_2_, 5 mM MnCl2, 3 mM Na_3_VO_4_, 1.25 mM DTT, and 20 mM ATP. After incubation for 2 hours at room temperature, the mixture was analyzed by Western blot using anti-phosphotyrosine (anti-p-Tyr) and anti-TRβ1 antibodies.

### Preparation of expression plasmids of TRβ1-ligand binding domain with mutations at Y321, Y395, Y406, and Y409 by phenylalanine (Phe)

The mammalian expression plasmids for the truncation mutants of TRβ1 were described previously [30]. We used the pcDNA3.1TRβ1ΔA/B/C (ie. ligand-binding domain LBD) as a template to construct expression vector that had mutation of phenyalaine (Phe) at Y321, Y395, Y406 and Y409, Mutagenesis was performed as described in the QuikChange II XL site-directed kit manual (catalog no. 200521; Stratagene, La Jolla, CA). The sequences of the forward and reverse primers were TRβ1Y321F forward, 5-GCTGCTGTGCGCTTTGACCC AGAAAGTGAG-3; TRβ1Y321F reverse, 5-CTCACTTT CTGGGTCA AAGCGCACAGCAGC-3; TRβ1Y395F forward, 5-GAGAGAATAGAAAAGT TCCAAGATAGTTTC-3; TRβ1Y395F reverse, 5-GAAACTATCTTGGAACT TTTCTATTCTCTC-3; TRβ1Y406F forward, 5-GCTGGCCTTTGAACAC TTTATCAATTACCG-3; TRβ1Y406F reverse, 5-CGGTAATTG ATAAAGTGTTCAAAGGCCAG C; TRβ1Y409F forward, 5-CACTATATCAATTTCCG AAAACACCACGTG-3; TRβ1Y409F reverse, 5-CACGTGGTGTTTTCGGAAATTGATATAGTG-3. Reverse and forward primers were complementary in sequence-covered mutation sites. The mutagenesis reaction was performed in a 25 ml volume using 50 ng template DNA (pcDNA3.1-FH-TRβ1 LBD) and 100 ng primers, and all other reagents were added following the QuickChange site-directed mutagenesis kit instructions. The cycling conditions were a 1-minute initial denaturation at 95°C, 18 cycles with 50 seconds denaturation at 95°C, 50 seconds annealing at 60°C, and 7 minutes extension at 68°C, and a final extension of 7 minutes at 68°C. The product was treated with 10 U DpnI and incubated for 1 hour at 37°C. Then, 2 ml DpnI-treated DNA was used for transformation of XL-10 Gold ultracompetent cells to select ampicillin-resistant colonies. The selected clones were verified by DNA sequencing.

### Preparation of expression plasmids of TRβ1 with tyrosine to phenylalanine mutations at Y321, Y395, Y406 and Y409

The mammalian expression vectors for full-length hTRβ1 mutants were constructed by using Flag-hemagglutinin tagged-pcDNA3.1-TRβ1 (pcDNA3.1-FH-TRβ1) as a template similarly as described above. The TRβ1 Phe mutants at Y321, Y395, Y406 and Y409 were prepared using the QuikChange II XL site-directed kit according to company instruction manuals. The primers of mutagenesis were the same as described for the mutations of the LBD described as above. The obtained mutants were verified by DNA sequencing.

### Generation of MDA cells stably expressing TRβ1 or TRβ1Y406F

Establishment of MDA-MB-468 cells stably expressing human TRβ1 (MDA-TRβ1 cells), TRβ1Y406F (MDA-TRβ1Y406F cells), or the control selectable marker Neo gene (Neo cells) was carried out similarly as described previously for HeLa cells [44]. Briefly, MDA-MB-468 cells were transfected with the expres­sion plasmid containing cDNA encoding Flag-hemagglutinin-TRβ1 (pcDNA3.1-FH-TRβ1), Flag-hemagglutinin-TRβ1Y406F (pcDNA3.1-FH-TRβ1Y406F), or the empty vec­tor containing only the cDNA for the selector marker, the Neo gene. After transfection, cells were selected with 200 μg/ml G418 (Invitrogen, Carlsbad, CA) for 2 weeks. G418-resistant colo­nies expressing TRβ1 and TRβ1Y406F were expanded for sub­sequent experiments. The expression of TRβ1 and TRβ1Y406F protein was verified by Western blot analysis using monoclonal anti-TRβ antibody (J53) [45].

### Western blot analysis and co-immunoprecipitation assays

The Western blot analysis was carried out as described by Furumoto *et al.* [46]. Anti-TRβ1 antibodies (C4; 1 μg/mL) [47] and J53 [45] were used at 2 μg/ml. The anti-p-Tyr (pY100, cat. #9411; 1:1000 dilution), p-Src (Tyr 416, cat. #2113; 1:1000 dilution), total-cSrc (cat. #2108, 1:1000 dilution), p-ERK (T202/Y204, cat. # 9101), total-ERK (cat. #9102) and and GAPDH (#2118) were purchased from Cell Signaling Technology. Anti-p-FAK (Y397, cat. 44624) and total FAK (cat. SC-557) were purchased from Invitrogen and Santa Cruz Biotechnology, respectively.

To demonstrate the phosphorylation of tyrosine of full-length and truncated TRβ1 in cells, expression plasmids for full-length TRβ1 (pcDNA3.1-TRβ1),Δ□□ TRβ1 (pcDNA3.1-Δ□□ TRβ1), ΔA/B/C TRβ1(pcDNA3.1-Δ□□C-TRβ1) were transiently transfected into MDA-MB-468 parent cells using Lipofectamine 2000 (Invitrogen, cat. 11668-027, Carlsbad, CA). Transfected cells were cultured in DMEM/F12 media including T3 deficiency FBS (Td) for 24 hours followed by without or with addition of 100 nM T3 for 15 min. Cellular extracts (1 mg) were prepared and immunoprecipitated with anti-Tyr antibodies followed by Western blot analysis using anti-TRβ1 (J53).

To determine the effect of SKI606 on the tyrosine phosphorylation of TRβ1 LBD and Y406F LBD mutant in cells, expression plasmid for TRβ1 LBD and Y406F LBD mutant were transiently transfected into MDA-MB-468 parent cells using Lipofectamine 2000 (Invitrogen, cat.11668-027, Carlsbad, CA). Transfected cells were cultured in DMEM/F12 media including T3 deficiency FBS (Td) for 24 hours followed by without or with addition of 0.5 μM or 1 μM SKI606 for 8 hours, and then treated without or with 100 nM T3 for 15 min before total protein extraction. Cellular extracts (2 mg) were prepared and immunoprecipitated with anti-Tyr antibodies followed by Western blot analysis using anti-TRβ1 (J53).

Co-immunoprecipitation of TRβ1 with cSrc was carried as described previously (Fozzatti PLOS one, 2013). Briefly, MDA-TRβ1, MDA-TRβ1Y406F, and Neo cells were treated in the absence or presence of T3 (100 nM) for 24 hours. Cell lysates (1 mg) were prepared and immunoprecipitated with monoclonal anti-TRβ1 antibody (C4; 4 μg), or control mouse anti-IgG antibodies (4 μg) followed by Western blot analysis using rabbit anti-cSrc antibodies (Cell Signaling, Cat# 2109, 1:1000 dilution).

### T3 binding assay

The TRβ1, TRβ1Y406F, and TRβ1PV proteins were prepared by *in vitro* transcription/translation (TNT-quick-couple transcription/translation system; Promega, cat. L1170) using plasmids pcDNA3.1 TRβ1, pcDNA3.1 TRβ1Y406F, and pcDNA3.1 TRβ1PV, respectively. *In vitro* translated proteins were analyzed by SDS-PAGE, followed by Western blotting using anti-TRβ1 antibody to ensure that equal amounts of receptor proteins were used in the binding assays. ([^125^I]-T3), 100μCi (3.7MBq, cat NEX110100UC) was purchased from PerkinElmer. The binding to TRβ1, TRβ1Y406F, and TRβ1PV was carried out by incubating with 0.2 nM [^125^I] T3 in the absence or increasing concentration of unlabeled T3 in 0.25 ml binding buffer for 90 minutes at room temperature. Protein bound [^125^I] T3 was separated from the unbound radioligand in a Saphadex G-25 (fine) column (5.5 × 1 cm) as described previously [48]. [^125^I]-T3 bound to receptor fractions was measured with a Gamma 5500B counter (Beckman Instruments, Inc., Fullerton, CA).

### Cell proliferation assay

The control (Neo), MDA-TRβ1, and MDA-TRβ1Y406F cells (5 × 10^4^ cells per well) were plated in 6-well plates (in triplicates) and cul­tured for 4 days in the presence or absence of T3 (100 nM). Cell proliferation was measured every 24 hours for 4 days using a cell counter (Beckmann Coulter, Indianapolis, IN) as described previously [25].

### Wound healing and invasion assay

Wound healing assay was carried out as previously described [39] with some modifications. The wound was applied with a pipette tip on the confluent cells, and nonattached cells were removed by gently flushing with fresh media. We visualized cell migration with an inverted microscope at ×100 Mag at every 6 hours for 24 hours. The cell migration was determined at the edges of the wound, and the percentage of migration was determined as the ratios between migrated distance and initial distance of the wound. For invasion assay, the method used was similar to the protocol described previously [49]. Invasion assay was performed in 8-μm-pore transwells (6.5 mm; Costar, Corning, NY) in quadruplicate. Transwells filters were layered with 100 μl of matrigel (BD Biosciences, cat. 356230) diluted 1:10 in PBS. After rinsing with PBS, cells were plated as above. Seventy-two hours later, cells migrating to the bottom of the filter were evaluated, after removal of material from the upper side of the filter, by 0.1% crystal violet staining and measurement of solubilized dye at A590.

### *In vivo* mouse xenograft study

The protocols for the use and care of the ani­mals in the present studies were approved by the National Cancer Institute Animal Care and Use Committee. Six-week-old female athymic NCr-nu/nu mice were obtained from the NCI-Frederick animal facility. The control MDA cells (Neo) and MDA-TRβ1 or MDA-TRβ1Y406F (5 × 10^6^ cells) in 200 μl sus­pension mixed with Matrigel basement mem­brane matrix (BD Biosciences, cat. 354234) were inoculated subcutaneously into the right flank of mice, similarly as previously described [25]. The tumor size was measured with calipers weekly until it reached ~2 cm in diameter. The mice were then sacrificed and the tumors dissected. The tumor volume was calculated as L × W × H × 0.5236.

### Statistical analysis

All data are expressed as mean ± the standard error of the mean (SEM). Significant differences between groups were calculated using Student’s t-test with the use of GraphPad Prism 5 (GraphPad Software, Inc., San Diego, CA). P<0.05 is considered statistically significant.

## References

[1] Cheng SY, Leonard JL, Davis PJ (2010). Molecular aspects of thyroid hormone actions. Endocrine reviews.

[2] Buzon V, Carbo LR, Estruch SB, Fletterick RJ, Estebanez-Perpina E (2012). A conserved surface on the ligand binding domain of nuclear receptors for allosteric control. Mol Cell Endocrinol.

[3] Johnson AB, O’Malley BW (2012). Steroid receptor coactivators 1, 2, and 3: critical regulators of nuclear receptor activity and steroid receptor modulator (SRM)-based cancer therapy. Mol Cell Endocrinol.

[4] Cao X, Kambe F, Moeller LC, Refetoff S, Seo H (2005). Thyroid hormone induces rapid activation of Akt/protein kinase B-mammalian target of rapamycin-p70S6K cascade through phosphatidylinositol 3-kinase in human fibroblasts. Molecular endocrinology.

[5] Moeller LC, Cao X, Dumitrescu AM, Seo H, Refetoff S (2006). Thyroid hormone mediated changes in gene expression can be initiated by cytosolic action of the thyroid hormone receptor beta through the phosphatidylinositol 3-kinase pathway. Nuclear receptor signaling.

[6] Furuya F, Hanover JA, Cheng SY (2006). Activation of phosphatidylinositol 3-kinase signaling by a mutant thyroid hormone beta receptor. Proceedings of the National Academy of Sciences of the United States of America.

[7] Leduc F, Brauch H, Hajj C, Dobrovic A, Kaye F, Gazdar A, Harbour JW, Pettengill OS, Sorenson GD, van den Berg A (1989). Loss of heterozygosity in a gene coding for a thyroid hormone receptor in lung cancers. American journal of human genetics.

[8] Sisley K, Curtis D, Rennie IG, Rees RC (1993). Loss of heterozygosity of the thyroid hormone receptor B in posterior uveal melanoma. Melanoma research.

[9] Chen LC, Matsumura K, Deng G, Kurisu W, Ljung BM, Lerman MI, Waldman FM, Smith HS (1994). Deletion of two separate regions on chromosome 3p in breast cancers. Cancer research.

[10] Gonzalez-Sancho JM, Garcia V, Bonilla F, Munoz A (2003). Thyroid hormone receptors/THR genes in human cancer. Cancer letters.

[11] Huber-Gieseke T, Pernin A, Huber O, Burger AG, Meier CA (1997). Lack of loss of heterozygosity at the c-erbA beta locus in gastrointestinal tumors. Oncology.

[12] Ali IU, Lidereau R, Callahan R (1989). Presence of two members of c-erbA receptor gene family (c-erbA beta and c-erbA2) in smallest region of somatic homozygosity on chromosome 3p21-p25 in human breast carcinoma. Journal of the National Cancer Institute.

[13] Joseph B, Ji M, Liu D, Hou P, Xing M (2007). Lack of mutations in the thyroid hormone receptor (TR) alpha and beta genes but frequent hypermethylation of the TRbeta gene in differentiated thyroid tumors. J Clin Endocrinol Metab.

[14] Ling Y, Xu X, Hao J, Ling X, Du X, Liu X, Zhao X (2010). Aberrant methylation of the THRB gene in tissue and plasma of breast cancer patients. Cancer Genet Cytogenet.

[15] Iwasaki Y, Sunaga N, Tomizawa Y, Imai H, Iijima H, Yanagitani N, Horiguchi K, Yamada M, Mori M (2010). Epigenetic inactivation of the thyroid hormone receptor beta1 gene at 3p24. 2 in lung cancer. Ann Surg Oncol.

[16] Li Z, Meng ZH, Chandrasekaran R, Kuo WL, Collins CC, Gray JW, Dairkee SH (2002). Biallelic inactivation of the thyroid hormone receptor beta1 gene in early stage breast cancer. Cancer Res.

[17] Martinez-Iglesias O, Garcia-Silva S, Tenbaum SP, Regadera J, Larcher F, Paramio JM, Vennstrom B, Aranda A (2009). Thyroid hormone receptor beta1 acts as a potent suppressor of tumor invasiveness and metastasis. Cancer research.

[18] Garcia-Silva S, Aranda A (2004). The thyroid hormone receptor is a suppressor of ras-mediated transcription, proliferation, and transformation. Molecular and cellular biology.

[19] Kim WG, Zhao L, Kim DW, Willingham MC, Cheng SY (2014). Inhibition of Tumorigenesis by the Thyroid Hormone Receptor beta in Xenograft Models. Thyroid: official journal of the American Thyroid Association.

[20] Guigon CJ, Cheng SY (2009). Novel non-genomic signaling of thyroid hormone receptors in thyroid carcinogenesis. Molecular and cellular endocrinology.

[21] Lemoine NR, Mayall ES, Jones T, Sheer D, McDermid S, Kendall-Taylor P, Wynford-Thomas D (1989). Characterisation of human thyroid epithelial cells immortalised *in vitro* by simian virus 40 DNA transfection. British journal of cancer.

[22] Kim DW, Zhao L, Hanover J, Willingham M, Cheng SY (2012). Thyroid hormone receptor beta suppresses SV40-mediated tumorigenesis via novel nongenomic actions. American journal of cancer research.

[23] Harvey R, Hehir KM, Smith AE, Cheng SH (1989). pp60c-src variants containing lesions that affect phosphorylation at tyrosines 416 and 527. Molecular and cellular biology.

[24] Parrilla R, Mixson AJ, McPherson JA, McClaskey JH, Weintraub BD (1991). Characterization of seven novel mutations of the c-erbA beta gene in unrelated kindreds with generalized thyroid hormone resistance. Evidence for two “hot spot” regions of the ligand binding domain. The Journal of clinical investigation.

[25] Park JW, Zhao L, Cheng SY (2013). Inhibition of estrogen-dependent tumorigenesis by the thyroid hormone receptor beta in xenograft models. American journal of cancer research.

[26] Wong CW, McNally C, Nickbarg E, Komm BS, Cheskis BJ (2002). Estrogen receptor-interacting protein that modulates its nongenomic activity-crosstalk with Src/Erk phosphorylation cascade. Proceedings of the National Academy of Sciences of the United States of America.

[27] Unni E, Sun S, Nan B, McPhaul MJ, Cheskis B, Mancini MA, Marcelli M (2004). Changes in androgen receptor nongenotropic signaling correlate with transition of LNCaP cells to androgen independence. Cancer research.

[28] Guo J, Wu HW, Hu G, Han X, De W, Sun YJ (2006). Sustained activation of Src-family tyrosine kinases by ischemia: a potential mechanism mediating extracellular signal-regulated kinase cascades in hippocampal dentate gyrus. Neuroscience.

[29] Furuya F, Shimura H, Yamashita S, Endo T, Kobayashi T Liganded thyroid hormone receptor-alpha enhances proliferation of pancreatic beta-cells. J Biol Chem.

[30] Dace A, Zhao L, Park KS, Furuno T, Takamura N, Nakanishi M, West BL, Hanover JA, Cheng S (2000). Hormone binding induces rapid proteasome-mediated degradation of thyroid hormone receptors. Proc Natl Acad Sci U S A.

[31] Anbalagan M, Huderson B, Murphy L, Rowan BG (2012). Post-translational modifications of nuclear receptors and human disease. Nuclear receptor signaling.

[32] Knutson TP, Lange CA (2014). Tracking progesterone receptor-mediated actions in breast cancer. Pharmacology & therapeutics.

[33] Edwards DP, Boonyaratanakornkit V (2003). Rapid extranuclear signaling by the estrogen receptor (ER): MNAR couples ER and Src to the MAP kinase signaling pathway. Molecular interventions.

[34] Lin KH, Ashizawa K, Cheng SY (1992). Phosphorylation stimulates the transcriptional activity of the human beta 1 thyroid hormone nuclear receptor. Proceedings of the National Academy of Sciences of the United States of America.

[35] Boonyaratanakornkit V, Scott MP, Ribon V, Sherman L, Anderson SM, Maller JL, Miller WT, Edwards DP (2001). Progesterone receptor contains a proline-rich motif that directly interacts with SH3 domains and activates c-Src family tyrosine kinases. Molecular cell.

[36] Fox EM, Andrade J, Shupnik MA (2009). Novel actions of estrogen to promote proliferation: integration of cytoplasmic and nuclear pathways. Steroids.

[37] Varricchio L, Migliaccio A, Castoria G, Yamaguchi H, de Falco A, Di Domenico M, Giovannelli P, Farrar W, Appella E, Auricchio F (2007). Inhibition of estradiol receptor/Src association and cell growth by an estradiol receptor alpha tyrosine-phosphorylated peptide. Molecular cancer research: MCR.

[38] Pena D, Pontillo C, Garcia MA, Cocca C, Alvarez L, Chiappini F, Bourguignon N, Frahm I, Bergoc R, Kleiman de Pisarev D, Randi A (2012). Alterations in c-Src/HER1 and estrogen receptor alpha signaling pathways in mammary gland and tumors of hexachlorobenzene-treated rats. Toxicology.

[39] Kim WG, Zhu X, Kim DW, Zhang L, Kebebew E, Cheng SY (2013). Reactivation of the silenced thyroid hormone receptor beta gene expression delays thyroid tumor progression. Endocrinology.

[40] Lu XL, Liu XY, Cao X, Jiao BH (2012). Novel patented SRC kinase inhibitor. Current medicinal chemistry.

[41] Liu ST, Pham H, Pandol SJ, Ptasznik A (2013). Src as the link between inflammation and cancer. Frontiers in physiology.

[42] Guigon CJ, Kim DW, Zhu X, Zhao L, Cheng SY Tumor suppressor action of liganded thyroid hormone receptor beta by direct repression of beta-catenin gene expression. Endocrinology.

[43] Wagner RL, Huber BR, Shiau AK, Kelly A, Cunha Lima ST, Scanlan TS, Apriletti JW, Baxter JD, West BL, Fletterick RJ (2001). Hormone selectivity in thyroid hormone receptors. Molecular endocrinology.

[44] Ying H, Furuya F, Zhao L, Araki O, West BL, Hanover JA, Willingham MC, Cheng SY (2006). Aberrant accumulation of PTTG1 induced by a mutated thyroid hormone beta receptor inhibits mitotic progression. The Journal of clinical investigation.

[45] Lin KH, Willingham MC, Liang CM, Cheng SY (1991). Intracellular distribution of the endogenous and transfected beta form of thyroid hormone nuclear receptor visualized by the use of domain-specific monoclonal antibodies. Endocrinology.

[46] Furumoto H, Ying H, Chandramouli GV, Zhao L, Walker RL, Meltzer PS, Willingham MC, Cheng SY (2005). An unliganded thyroid hormone beta receptor activates the cyclin D1/cyclin-dependent kinase/retinoblastoma/E2F pathway and induces pituitary tumorigenesis. Mol Cell Biol.

[47] Bhat MK, McPhie P, Ting YT, Zhu XG, Cheng SY (1995). Structure of the carboxy-terminal region of thyroid hormone nuclear receptors and its possible role in hormone-dependent intermolecular interactions. Biochemistry.

[48] Kitagawa S, Obata T, Hasumura S, Pastan I, Cheng SY (1987). A cellular 3,3′,5-triiodo-L-thyronine binding protein from a human carcinoma cell line. Purification and characterization. The Journal of biological chemistry.

[49] Yu Y, Merlino G (2002). Constitutive c-Met signaling through a nonautocrine mechanism promotes metastasis in a transgenic transplantation model. Cancer research.

